# Analysis of Sclerotia-Associated Fungal Communities in Cool-Temperate Forest Soils in North Japan

**DOI:** 10.1264/jsme2.ME14135

**Published:** 2015-02-04

**Authors:** Anzilni F. Amasya, Kazuhiko Narisawa, Makiko Watanabe

**Affiliations:** 1 Department of Geography, Tokyo Metropolitan UniversityHachioji-shi, Tokyo 192–0397Japan; 2 Department of Bioresource Science, College of Agriculture, Ibaraki UniversityAmi-machi, Ibaraki 300–0393Japan

**Keywords:** sclerotia, *Cenococcum geophilum*, T-RFLP

## Abstract

We herein investigated sclerotia that were obtained from cool-temperate forests in Mt. Chokai and Mt. Iwaki in north Japan and tentatively identified as the resting bodies of *Cenococcum geophilum*. The profiles of sclerotia-associated fungal communities were obtained through T-RFLP combined with clone library techniques. Our results showed that sclerotia in Mt. Chokai and Mt. Iwaki were predominated by *Arthrinium arundinis* and *Inonotus* sp., respectively. The results of the present study suggested that these sclerotia-associated species were responsible for the formation of sclerotia or sclerotia were originally formed by *C. geophilum*, but were subsequently occupied by these species after *C. geophilum* germinated or failed to survive due to competition.

Fungal species may respond to environmental stress such as desiccation by hardening their mycelia and forming a compact mass with the appearance of resting structures, which are referred to as sclerotia. One of the most common soil fungal species that forms sclerotia is *Cenococcum geophilum*, which is distributed worldwide (6, 13) and has more than 200 species of host plants (18). The sclerotia of *C. geophilum* are abundant and have been examined in many parts of the world. The sclerotia of *C. geophilum* biomass was previously estimated to be 440 kg ha^−1^ in old-growth Norway spruce forests in south Sweden (4) and 2785 kg ha^−1^ in a second-growth Douglas-fir stand in the Oregon Coast Range (8). The distribution of *C. geophilum* sclerotia has also been examined in the forest soils of the Harz Mts. in Germany (28, 34). The sclerotia of *C. geophilum* are distributed in volcanic ash soils in central Japan (25, 32, 34) and Andosol profiles in central Japan (33), and were also found to be abundant in *Pinus thurnbergii* in the coastal pine forests of Japan (20). These studies “tentatively identified” sclerotia as the resting bodies of *C. geophilum* according to a description of its morphological characteristics provided by Trappe (31) and Kumada and Hurst (16) without further molecular identification studies; however, a broad range of fungal species have the ability to form sclerotia. Limitations have been associated with the identification of sclerotia mainly because they grow slowly in cultures (2) and are difficult to isolate in axenic cultures (5). Furthermore, sclerotial walls are difficult to extract because they contain a non-hydrolyzable residue consisting of a highly resistant melanin-like pigment, which plays an important role in the resistance of sclerotia towards chemical and biological degradation (3). On the other hand, sclerotia accumulate relatively high concentrations of carbohydrates, fats, and proteins during their growth, which may provide good nutritional niches for the development of other microorganisms associated with sclerotia (36). These sclerotia-associated microorganisms provide valuable information for studies on microbial diversity in the rhizosphere, biocontrol for plant pathogens (38), and also functional heterogeneity in adaptations towards drought (13). Since sclerotia are formed from fungal species, analyzing sclerotia-associated fungal communities in sclerotia may confirm the identification of “tentatively-identified as *C. geophilum*” sclerotia based on the fungal community profiles. One of the most widely used community profiling approaches is the Terminal Restriction Fragment Length Polymorphism (T-RFLP) analysis (17), which provides a simple means to assess changes in microbial community structures on temporal and spatial scales by monitoring the gain or loss of specific fragments from the profiles (9, 19, 22). When coupled with 16S rRNA clone library construction and clone sequencing, additional specific information on the composition of microbial communities can be obtained (29). Since no cultivation required, this combined technique may offer a rapid solution for identifying sclerotia by analyzing the fungal communities associated with sclerotia. In order to yield abundant sclerotia, we selected forest soils in the cool-temperate forests of Japan. The parent materials of the soils were weathered Andesite associated with non-tephric loess deposits transported from continental China (32). Its extremely high precipitation and non-andic mineralogical properties regulated the low pH values and high content of exchangeable aluminum in soils, which consequently influenced the development of sclerotium grains (33). Therefore, the aim of the present study was to identify sclerotia by analyzing sclerotia-associated fungal communities collected from cool-temperate forests in Japan using a modified sclerotia extraction method followed by T-RFLP combined with a clone library analysis.

Sclerotia were collected from Mt. Chokai in Yamagata Prefecture (39°6′42.53″ N, 139°58′0.61″ E, site elevation: 730 m; annual rainfall: 2,362 mm), and Mt. Iwaki of Aomori Prefecture (40°39′0.37″ N, 140°16′33.60″ E, site elevation: 790 m; annual rainfall: 1,750 mm), which are both in north Japan. Vegetation at the site in Mt. Chokai (Cambic Podzols, WRB/FAO-Unesco) was predominated by *Fagus crenata*, and less dominant plants were *Viburnum furcatum* and *Lindera umbellata*, while the forest floors were dominated by *Sasa kurilensis*. The site at Mt. Iwaki (Dystric Cambisols, WRB/FAO-Unesco) mostly consisted of *Quercus serrata*, *Fagus crenata*, and *Betula ermanii* trees and *Sasa kurilensis* dominated the forest floors. Nine points were selected evenly within a 10×10 m^2^ quadrat. Each point was a 20×20 cm^2^ square in which litter, F, and H layers were removed, and A horizons were collected using 2 cylinders each with a volume of approximately 800 cm^3^. Sclerotia were handpicked from the A horizons and the surface of sclerotia was then sterilized using the technique described by Ohta *et al.* (25). Every grain was washed with 1 mL of sterile water in a microtube for 1 min with vortex mixing, the washing solution was then removed from the tube with a sterile pipette, and this procedure was repeated ten times. Surface-sterilized sclerotia were weighed to approximately 100 mg for each studied area and placed in a sterile 2-mL centrifuge tube. A metal crusher was inserted into the tube and 200 μL of Lysis Buffer (10 μL Tris-Cl pH 8 1 M; 2 μL EDTA 0.5 M; 10 μL Proteinase K; 100 μL SDS 10%, and 878 μL H_2_ O) was added and homogenized. The crushed sclerotial solution was incubated at 37°C with shaking for 2 h. The lysed solution was extracted using phenol/chloroform extraction, followed by the addition of Binding buffer (60 gr Guanidine Thiocyanate; 10 mL Tris-HCl 1 M; and 40 mL distilled water) and silica. Guanidine thiocyanate was washed out using the Wash Buffer (10 mM Tris-HCl pH 7.5; 100 mM NaCl:Ethanol=1:4). PCR was conducted using the universal primers ITS1F and ITS4 (10) with the iProof™ High-Fidelity PCR kit (Bio-rad Laboratories, Hercules, CA, USA) under a hot start at 98°C for 30 s, then 35 cycles consisting of 10 s at 98°C, 30 s at 58°C, and 30 s at 72°C in a DNA thermal cycler (Takara Bio, Otsu, Japan). The PCR products were purified from the Seakem^®^ GTG™ Agarose gel and continued with the silica method. The purified PCR products were then amplified through a quenching PCR using the quenching-fluorescence-labeled primers qLR21 and ITS1F. Thirty microliters of the qPCR reaction mixture was prepared by adding 0.1 μg template sclerotial DNA, 1.0 μL of 10 pmol μL^−1^ primers, Takara Ex Taq™ dNTPs, and 3 μL of optimized 10xEx buffer (Takara Bio) in a PCR cycler. The PCR for T-RFLP profiling was conducted after denaturing for 2 min at 98°C, followed by 30 cycles consisted of 30 s at 95°C, 45 s at 54°C, and 90 s at 72°C. Aliquots of the amplified fragments were then separately digested with the restriction enzymes AluI, HhaI, and HaeIII (Takara Bio) according to the manufacturer’s instructions. The lengths of T-RFs from the amplified fragments were determined with a 3130xl DNA Sequencer (Applied Biosystems, Foster City, CA, USA) by mixing 2 μL of purified T-RF DNA with 15 μL of Hi-Di formamide and 0.1 μL of DNA standard LIZ^®^600 (Applied Biosystems). The procedure was followed by denaturing at 96°C for 2 min and immediately chilled on ice ahead of electrophoresis using an ABI automated sequence analyzer. The lengths of the fluorescently labeled T-RFs were determined after electrophoresis by comparisons with internal standards using GeneMapper^®^ software (version 3.7, Applied Biosystems). T-RFLP profiling of the fungal communities in sclerotia resulted in peaks ranging from base sizes of 50–650 base pairs. The dominant T-RF peaks were determined by an analysis of clone libraries. T-RF solutions were ligated using the pGEM^®^-T Easy Vector (Promega, Madison, WI, USA), and *Escherichia coli* DH5α High Efficiency Competent Cells was used as hosts for recombinant plasmids and grown at 37°C in LB agar, to which 100 μg mL^−1^ of ampicillin, IPTG, and Xgal to a final concentration of 40 μg mL^−1^ had previously been added. White colonies were selected and sequences were determined using the BigDye^®^ Terminator v3.1 Cycle Sequencing Kit (Applied Biosystems) and read on an Applied Biosystems 3130xl Genetic Analyzer. The primer M13 Primer RV was used in sequencing reactions to obtain partial DNA sequences. The DNA sequences were aligned using MEGA version 5 (30), and all sequences determined were compared to NCBI databases using the BLAST program.

Sclerotia from Mt. Chokai were spherical, gray-brownish-black, and relatively small, with a diameter range between 0.4–2.6 mm (average 1.33±0.06 mm; *n*=54), while sclerotia in Mt. Iwaki were not all perfectly round, their color was shiny-black, and they had a relatively large diameter, with a range between 0.8–4.4 mm (average 2.73±0.11 mm; *n*=54). Based on their morphological characteristics, sclerotia found in both areas corresponded to most of the descriptions reported by Trappe (31), which were a diameter of 0.05–4 mm or greater, a jet-black color, hard, smooth, and mostly spherical. The average sizes of sclerotia collected from Mt. Iwaki were significantly larger than those collected from Mt. Chokai (*p*<0.01 by the Student’s *t*-test). According to Matsumoto and Tajimi (21), the size of sclerotia responds to fungal strategies to adapt to environmental changes, whereas certain species in areas in which it is difficult to forecast environmental changes form slightly smaller sclerotia with less reserve substances in the active term. Therefore, the smaller size of sclerotia in Mt. Iwaki may indicate that this area experienced fewer environmental changes than Mt. Chokai. A larger sclerotial diameter may also indicate a high Al_EX_ content in the soil or exposure to an event that enriched Al_EX_ (27), which was observed through the lower pH in the soils of Mt. Iwaki than those of Mt. Chokai. The number of sclerotia ranged between 15 and 27 grains (average 19.83±0.81) per 800 cm^3^ in Mt. Chokai, while Mt. Iwaki sclerotia were more abundant, ranging between 12 and 47 grains (average 33.56±2.37) per 800 cm^3^. Sclerotia in Mt. Iwaki also had a higher count density, weight, and weight density than those of Mt. Chokai (Table 1). This result was consistent with the findings of Watanabe *et al.* (33) in which the development of sclerotia was influenced by high active aluminum in soils with low pH. Poor results were obtained when sclerotial DNA was extracted with a commercially available DNA extraction kit. On the other hand, the lysis buffer continued with phenol/chloroform extraction followed by the binding buffer and silica method used in this study led to more stable results, but was relatively time consuming. The fungal community profile of sclerotia from Mt. Chokai showed one dominant peak (Fig. 1), which was identified as *Arthrinium arundinis* (Ascomycota: Sordariales), a species described as dematiaceous or having dark-walled septate hyphae hyphomycetes (26). Species of Sordariales were also abundant and commonly detected in sclerotia collected from forests dominated by *Quercus* and *Pinus* in Florida and Georgia, USA (24), and have been recognized as one of the soil fungal species responsible for bamboo degradation (14). Nakashizuka (23) reported that the survival rate of *Fagus crenata* seedlings on the floor where dwarf bamboo had withered was markedly higher than that on the floor where dwarf bamboo survived. Moreover, the removal of understory dwarf bamboo increased the net carbon gain and transpiration rates of overstory trees (15); therefore, the removal of dwarf bamboo in relatively young stands may greatly enhance the productivity of overstory trees in the long-term (12). Based on these findings, the presence of *A. arundinis* in sclerotia from the beech forest floor in Mt. Chokai is suggested to play an important role in the survival of *F. crenata*. *Hypocrea lixii*, which was found in sclerotia from both of the sites examined, is also known to be the sexual reproductive stage or teleomorph of *Trichoderma harzianum* (1), a fast-growing soil fungal species reported to be effective in the biocontrol of plant-pathogenic fungi and soil-borne diseases (35). The presence of *H. lixii* in sclerotia was not considered to be a contaminant from the sclerotial surface because, according to Elad *et al.* (7), the mycoparasitic species of *Hypocrea/Trichoderma* degrade and grow within the resting structures (sclerotia) that are produced by a wide variety of pathogenic fungi, such as *Sclerotinia* spp., *Typhula* spp., *Macrophomina phaseolina*, and *Verticillium dahliae*. The results obtained from Mt. Iwaki showed one major peak, which was identified as *Inonotus* sp. (Basidiomycota: Hymenochaetales), a fungus that has been identified as one of the common diseases in birch trees, including *Betula ermanii*. According to Yamaguchi (37), the white-rot fungus, *I. obliquus*, the casual microorganism of canker disease in Japanese birch, has been found in Honshu and northern Japan, especially in Hokkaido. Hattori isolated *I. obliquus* in the sclerotial tissue of *B. platyphylla* Sukachev var. japonica (Miq) Hara in Tochigi, North Japan (Hattori, T. 1990. National Institute of Agricultural Sciences (NIAS) Genebank; http://www.gene.affrc.go.jp/databases-micro_search_detail_en.php?maff=420279). This finding suggested that the isolation of *Inonotus* sp. from the sclerotia of Mt. Iwaki may serve as a pathogen for the birch trees observed in the studied site. *Phyllactinia* sp. is a fungus from Ascomycota that causes a powdery mildew on leaves and stems on a broad range of host plants, including *Quercus serrata* (11). Based on the fungal community profiles of sclerotia, *C. geophilum* was not detected in either site. The T-RFLP method is capable of detecting the presence of a species, but cannot unequivocally indicate the absence of a species (5). Therefore, it is possible that other species, including *C. geophilum*, in sclerotia may have existed, but failed to be sufficiently amplified for detection. However, the results of our study suggest that: (i) the sclerotia observed were formed by *A. arundinis* in Mt. Chokai and by *Inonotus* sp. in Mt. Iwaki; or (ii) sclerotia were originally formed by *C. geophilum*, but were subsequently occupied by other species after *C. geophilum* germinated or failed to survive due to competition with other fungal species. The latter has been supported by Obase *et al.* (24) who demonstrated that sclerotia-associated fungi may be specialized mycoparasites or saprobes that preferentially decay fungal tissues or act as endophytes that colonize *Cenococcum* sclerotia without any aggressive interactions. However, given the low success rate of the isolation of *Cenococcum* (24) and its absence in our study, we cannot rule out the possibility that the sclerotia observed were formed by *A. arundinis* in Mt. Chokai and by *Inonotus* sp. in Mt. Iwaki. Further studies need to be conducted in order to confirm either suggestion.

We herein demonstrated that the fungal community profiling of sclerotia was an effective approach for identifying sclerotia, and also contributed to broadening our understanding of the roles and functions of sclerotia in forest soil.

## Figures and Tables

**Fig. 1:**
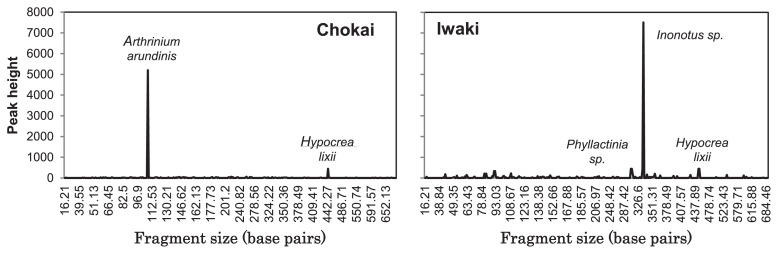
T-RFLP profile of sclerotia from Mt. Chokai (left) and Mt. Iwaki (right) using restriction enzyme HhaI.

**Table 1:** Soil and sclerotial properties.

*Soil*	Mt. Chokai	Mt. Iwaki
Litter weight (g)	77.67±13.49	47.89±6.71
Weight of F and H Horizons (g)	287.44±32.93	464.78±45.58
O Horizon thickness (cm)	5.08±0.36	5.31±0.03
Bulk density of A horizon (g cm^−3^)	0.45±0.04	0.35±0.04
Soil pH (H_2_ O)	4.60±0.09	3.96±0.04
Soil pH (KCl)	3.88±0.04	3.44±0.06

*Sclerotia*	Mt. Chokai	Mt. Iwaki

Count density (grains g^−1^)	0.14±0.06	0.16±0.03
Mean grain weight (mg)^a^	0.83±0.04	1.80±0.05
Weight density (mg g^−1^)^a^	0.11±0.02	0.29±0.04

Data are mean ± standard errors from 9 repeats for soils and 18 repeats for sclerotia.

a *p*<0.01 by the Student’s t-test.
